# Double-deep Q-learning to increase the efficiency of metasurface holograms

**DOI:** 10.1038/s41598-019-47154-z

**Published:** 2019-07-29

**Authors:** Iman Sajedian, Heon Lee, Junsuk Rho

**Affiliations:** 1 Department of Mechanical Engineering, Pohang University of Science and Technology (POSTECH), Pohang, 37673 Republic of Korea; 2 Department of Materials Science and Engineering, Korea University, Seoul, 02842 Republic of Korea; 3 Department of Chemical Engineering, Pohang University of Science and Technology (POSTECH), Pohang, 37673 Republic of Korea

**Keywords:** Nanophotonics and plasmonics, Metamaterials, Applied mathematics, Computational science

## Abstract

We use a double deep Q-learning network (DDQN) to find the right material type and the optimal geometrical design for metasurface holograms to reach high efficiency. The DDQN acts like an intelligent sweep and could identify the optimal results in ~5.7 billion states after only 2169 steps. The optimal results were found between 23 different material types and various geometrical properties for a three-layer structure. The computed transmission efficiency was 32% for high-quality metasurface holograms; this is two times bigger than the previously reported results under the same conditions. The found structure is transmission-type and polarization-independent and works in the visible region.

## Introduction

Metasurfaces can manipulate the phase and spectrum of impinging light^1–8^. The geometrical properties of metasurfaces can be tuned to change the phase of light for desired applications. Many applications have emerged from this idea, including ultrathin lenses^9,10^, vortex beam generators^11,12^ and holograms^13–21^. Different designs have been proposed to increase the holograms’ efficiency^18–21^; some used metallic structures but the efficiency in visible wavelengths was low because of metals’ intrinsic loss^22–25^. Some efficient designs work only for reflected light^26^.

The incident light’s polarization must be considered when a metasurface hologram is designed. Some of the proposed metasurfaces are noncircular^18,27–29^ so they only work for a specific polarization. Some are highly efficient and work independently of polarization, but they do not work in visible light^20^. The ideal structure would generate the whole phase map, work with transmitted light, work in the visible regime, be independent of polarization, and have high efficiency. No structure has yet satisfied all these conditions simultaneously.

The task of finding the best geometrical parameters and choosing the right materials for a structure is always a challenge. Recently, researchers have used neural networks (NNs) to design nanophotonic structures^30–36^, and to design chiral metamaterials^37^. The double deep Q-learning network (DDQN)^38^ has been used to find the optimized parameters for a photonic structure^39^. Here we use DDQN to optimize the design of a metasurface holograms. This method is like an intelligent sweep, which learns how to efficiently explore the given parameter space to reach the highest reward in the lowest time, and it can be used to optimize a given physical structure.

We would like to clarify why using DDQN (which belongs to the family of reinforcement learning (RL) methods) is much more efficient (or probably the only way) than using classic optimization methods like Monte-Carlo search, swarm intelligence, genetic algorithms or Bayesian method as a few examples. In neural networks, a number of hidden layers of nodes connect the input data to the output data. This connection is then optimized by an optimization method. This way the network not only optimizes the problem but also *learns* from it. To compare this to a human mind, assume that we are getting a reward for each step that we take. For example if we get 10 points for the first step and 20 points for the second step, we immediately *learn* that to get to 100 points we need to take 10 steps, and no further actions is needed to be taken. In a same way, an RL method optimizes a problem by learning from it and not just finding a maxima or minima in a mathematical space (like optimization methods). This leads to a much more efficient optimization method and in some cases the only method that can find the solution for a complex problem.

A detailed benchmark of DDQN compared to other methods is provided by other researchers^38^. We explained the details of this method and how it can be used in optics and its benefits previously^39^. Reinforcement learning was also used for identifying variational protocols in quantum physics^40^ as an optimizing mechanism.

## Methods

We can tune the phase of the incident light by changing the geometrical properties of the metasurfaces. The metasurface consists of nano-antennas with dimensions much smaller than the operating wavelength, so the thin layer of metasurface acts as a homogeneous medium with different refractive index *n* than the surrounding medium. The function of this thin layer is to apply a small phase delay to the light that is passing through it^26^. *n* of this thin layer can be controlled by changing the geometrical properties of the nano-antennas; the change in *n* leads to different phase delays by different structures. So each structure creates a phase delay. This way we can create a phase map (which is a collection of different phase delays from −π to π) by combining different structures.

Some authors use non circular Nano-antennas (like V-shaped^25^, rectangular^22^,…) to produce the required phase map. As an example, V-shaped Nano-antennas can easily be tuned by changing the length of each antenna or by changing the angle between them, to create the required phase delay. But they only work for a specific polarization. A structure that can produce a polarization independent phase delay should be cylindrical. But the problem with cylindrical structures is that they can only be tuned by their radius to generate the required phase delay, compared to non-circular shaped structures (since the thickness should be kept constant for manufacturing limitations). This makes it very hard to find the right structure which can generate the whole phase map and at the same time have a high transmission efficiency. Adding lattice constant and material type variables (which is common between all holograms) to this problem makes it very hard for human researchers to check all the possibilities to find the optimum structures. Here we use an AI code to help us find the optimum structure.

### Structure definition

**Figure 1:**
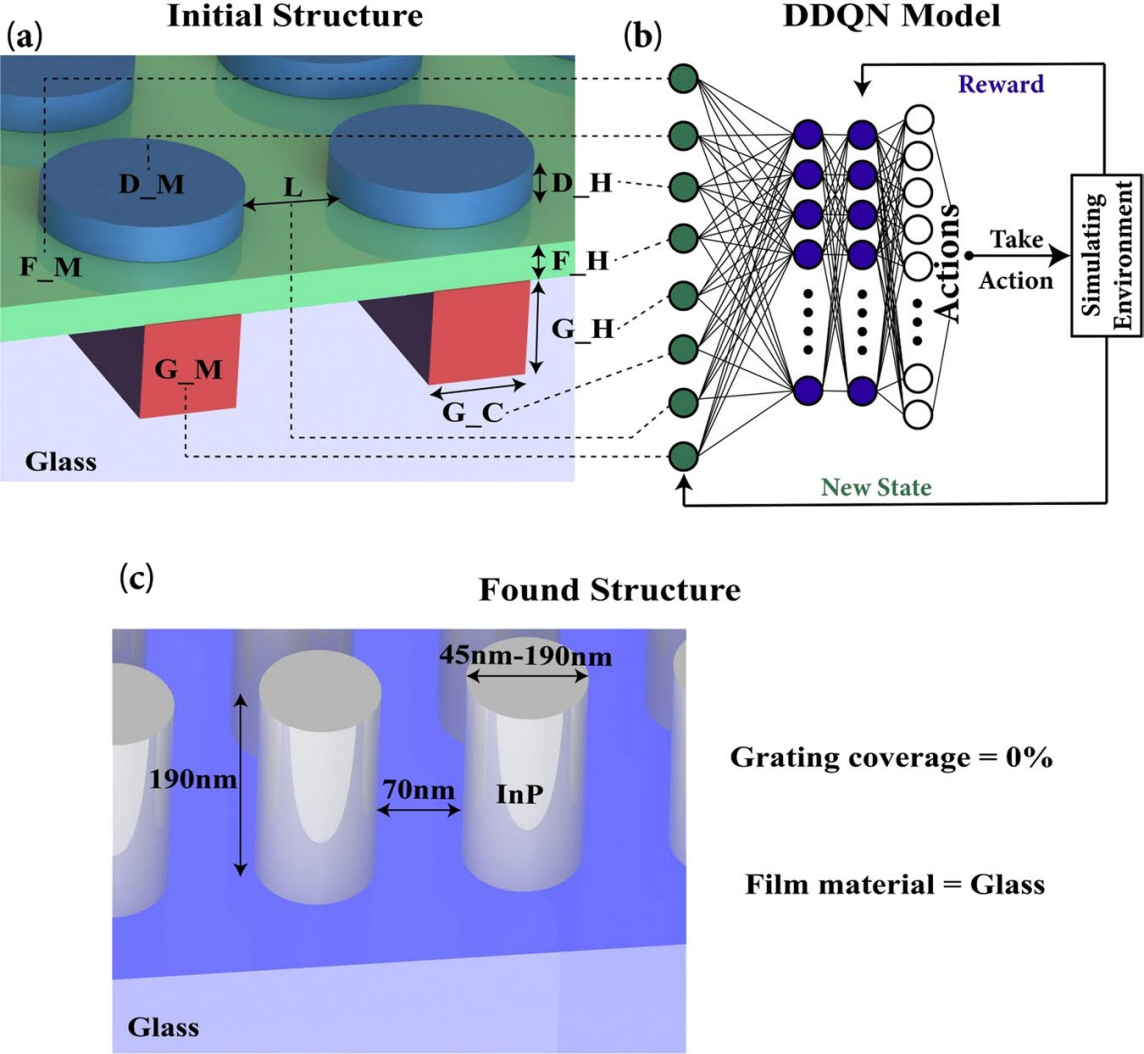
Schematic of application of DDQN algorithm to find high-efficiency holograms. (**a**) Initial structure used as the input DDQN at each step. Abbreviations are defined in the text and in Table 1. (**b**) Structure of DDQN method used to optimize the metasurface hologram. (**c**) The optimal structure found by DDQN with a high efficiency for generating holograms. The DDQN determined that no grating or film is required, by setting the coverage of the grating to zero and the material of the film as glass.

Here we try to find the optimal structure type to achieve high efficiency in the visible range for transmission-type holograms. The metasurface structure that we chose for this idea is a nano-disk laying on a thin film that is laying upon a grating, all on a glass substrate (Fig. 1(a)). Having this structure as the starting point covers many possibilities. The combination of the grating with nano-disks can increase transmission in metallic metamaterials by forming a structure that is similar to a Fabry-Perot cavity^41^. Using the circular shape for nanoantennas makes the hologram independent of polarization. All geometrical properties (except disk radius) and material types will be found using the DDQN. The important factor here is that the DDQN decides to use the starting structure as it is or to change it (removing grating, thin film, or both).

### DDQN structure

DDQN can be used to optimize a physical structure, as described previously^39^. Briefly, based on the given and future rewards, DDQN tries to connect the state of the structure to the action that should be taken (Fig. 1(b)). In DDQN we have two neural network models. A main model and an auxiliary model. The auxiliary model is used to update the main model’s weights, and the main model is used to predict the actions. These models had 3 hidden layers with 24, 48, 24 neurons each, with an Adam optimizer with a learning rate of 0.005^39^. A Markov decision process^42^ is used to predict the actions.

The model creates some data for itself by initial guessing at the beginning and by doing some actions (the model creates some data by itself from what it learned so far) as the code progresses, and all of these data are saved as an experience replay. This experience replay keeps getting updated as the model progresses (the old data is replaced by new data) so the model learns from the newly generated data. In other words, the model is training on data that is continuously updated.

**Figure 2:**
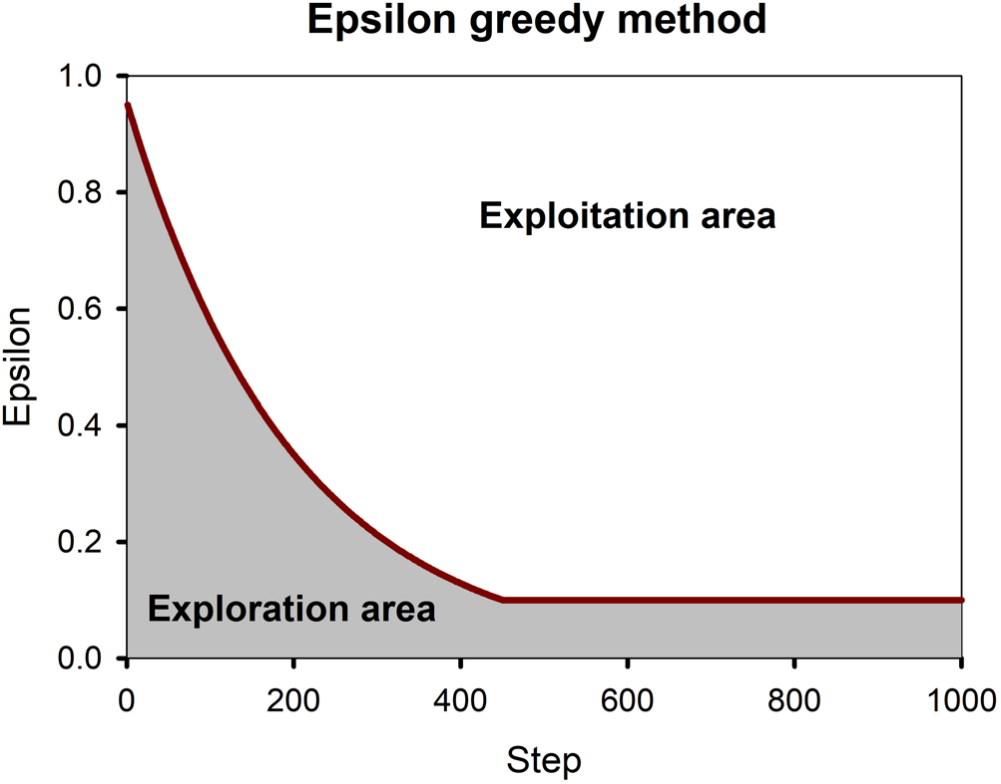
Epsilon greedy method. At each step, a random number is generated by the model. If the number was lower than epsilon in that step (exploration area) the model chooses a random action and if it was higher than epsilon in that step (exploitation area) the model chooses an action based on what it learned.

An epsilon-greedy method is used to create the initial database. This method determines when the guessing should finish and the learning should start. To do this we define an epsilon function starting from 0.95 to 0.1 with a decay rate of 0.995 as shown in Fig. 2. At each step, a random number is generated by the code. If the generated random number was lower than epsilon, then the model guesses the next action randomly (known as exploration), and if it was higher than epsilon the model predicts the next action by what it learned so far. At each step, the epsilon decays until it reaches 0.1 (this assures that the model always has a 10 percent chance of exploration).

At each step, the DDQN changes a geometrical property or material type of the structure. Based on the given feedback from the simulating environment it learns the effect of the change it made, and so it learns how to act better in future. The model consists of three parts: (1) the state of the structure at each step; (2) the action that should be taken to change the geometrical properties of the structure at each step; (3) a reward system that awards or penalizes the model for the action that it chose.

The state of the structure is composed of the geometrical properties and material types of the structure at each step:

- Nano-disk material type (D_M): 23 different materials.
- Thin film material type (F_M): 23 different materials.
- Grating material type (G_M): 23 different materials.
- Nano disk thickness (D_H): 10 nm–250 nm, step size: 10 nm; number of steps: 24
- Film thickness (F_H): 10 nm–150 nm, step size: 10 nm; number of steps: 14
- Grating thickness (G_H): 10 nm–150 nm, step size: 10 nm; number of steps: 14
- Grating coverage (G_C) in each unit cell: 0–100%; number of steps: 10
- The spacing between disks (L): 20 nm–120 nm, step size: 10 nm; number of steps: 10

The total number of *possible states = 23 × 23 × 23 × 24 × 14 × 14 × 10 × 10 = 5,723,356,800*.

We did not include the disk’s radius in the parameters, because it is used to evaluate the structure’s ability to produce the needed phase map. At each state, a separate loop is performed on the disk’s radius between 45 nm to 190 nm and the phases generated by different radii are computed and saved. If the phase range generated by the structure is big enough for holographic uses, the structure is considered as a candidate for optimization by the DDQN. All of these processes are performed in the reward system.

### Action definitions

The next step is to define the actions, which determine what the model should do at each step. To change the material of the disks, film, and grating we represented 23 materials in a matrix (Table 1). If the model wants to change the material of one of the parts, it simply changes the index of the material matrix of that specific part. Two actions are defined for changing the material of each part: one to increase the material’s matrix index and one to decrease it. The definitions of actions are shown in Table 2.

**Table 1:** List of materials used for disks, thin film, and grating.

ID#	Material	ID#	Material
1	Ag	13	Ta
2	Al	14	Ti
3	Au	15	TiN
4	Cr	16	W
5	Cu	17	GaAs
6	Fe	18	InAs
7	In	19	InP
8	Ni	20	Ge
9	Pd	21	Si
10	Pt	22	Si_3_N_4_
11	Rh	23	SiO_2_
12	Sn

**Table 2:** Definitions of actions used in DDQN.

Action No.	Action Definition
0	Decrease the spacing between disks (L) by 10 nm. (min 20 nm)
1	Increase the spacing between disks (L) by 10 nm. (max 120 nm)
2	Decrease the grating thickness (G_H) by 10 nm. (min 10 nm)
3	Increase the grating thickness (G_H) by 10 nm. (max 150 nm)
4	Decrease the disk thickness (D_H) by 10 nm. (min 10 nm)
5	Increase the disk thickness (D_H) by 10 nm. (max 250 nm)
6	Decrease the film thickness (F_H) by 10 nm. (min 10 nm)
7	Increase the film thickness (F_H) by 10 nm. (max 150 nm)
8	Decrease the grating coverage (G_C) by 10%. (min 0%)
9	Increase the grating coverage (G_C) by 10%. (max 100%)
10	Decrease the material ID of disks (D_M) by 1. (min 1)
11	Increase the material ID of disks (D_M) by 1. (max 23)
12	Decrease the material ID of the film (F_M) by 1. (min 1)
13	Increase the material ID of the film (F_M) by 1. (max 23)
14	Decrease the material ID of the grating (G_M) by 1. (min 1)
15	Increase the material ID of the grating (G_M) by 1. (max 23)

### Reward system

The final step is to define a reward system. It gives feedback to the model at each step, so it learns to improve its actions in future steps. We designed the reward system to give the highest feedback to the phase-generating property of the structure, and lesser feedback to its efficiency; i.e., the model prioritizes the structures that increase the phase map, then considers their efficiencies. To do this we divided the range of −π to π into six equal parts. A model gets 100 points for finding a structure that generates one of these parts, so in total, a model can get 600 points for finding a structure that can generate the whole phase map. A model gets additional points for the minimum transmitted power of the structure times 100. For example, if a structure generates four phase parts and has a minimum transmitted power of 0.25 it will get 4 × 100 + 0.25 × 100 = 425 points. This way, a structure that generates a large number of phase parts will be preferred by the model compared to a structure with a lower number of generated phase parts regardless of the structure’s efficiency. We choose this scheme because we seek a structure that can generate the whole phase map. The scheme also sets the terminating reward of the structure as 700 as is needed for DDQN model. A score of 700 means that the model has reached its ideal structure and should stop looking for new structures. The final found structure by DDQN is shown in (Fig. 1(c)).

### Generating the hologram

**Figure 3:**
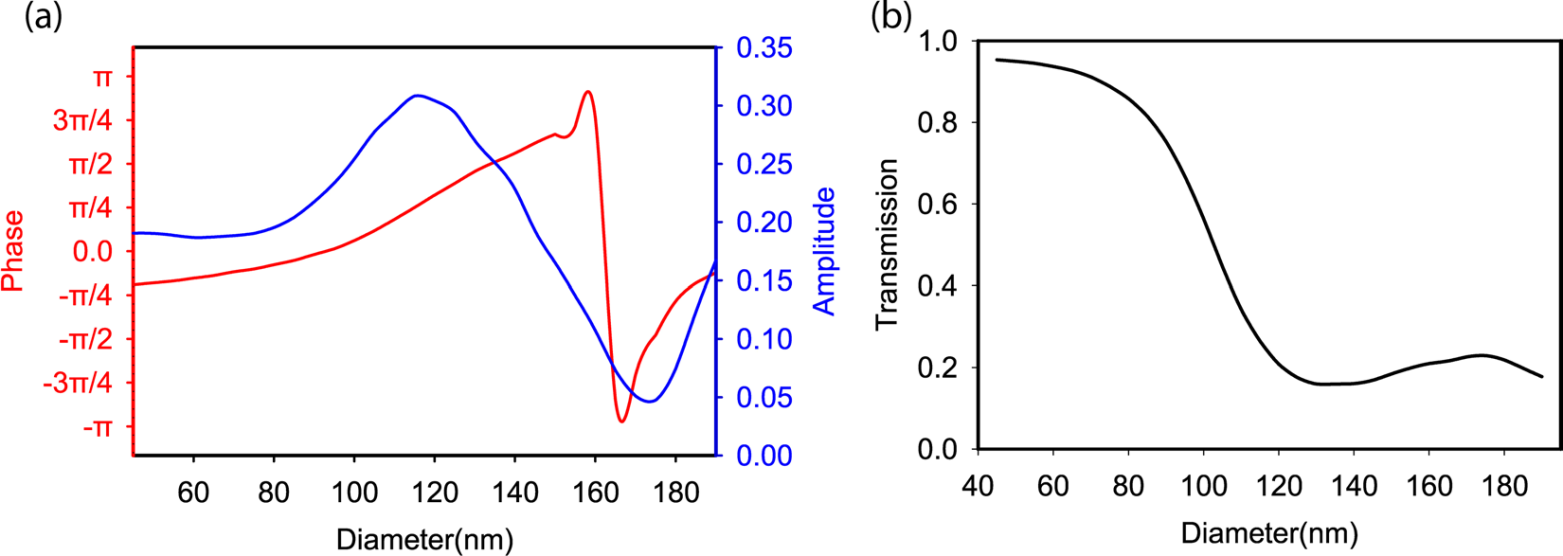
Physical results of the optimal structure found by DDQN. (**a**) Phases and amplitudes of the S-parameter for transmitted light generated by disks with different radii. This information is used to create the phase map needed for generating the hologram. (**b**) Effect of disk radius on transmitted power, which is used to determine the hologram’s efficiency.

Now we discuss how the found structure can be used to generate holograms. The first step is to find the phase that the structure generates. This procedure is performed by calculating the S-parameter, which shows the generated phase. We generated the whole phase range of [−π, π] while changing the radius of the disk from 45 nm to 190 nm. The radius affected the phase and amplitude of the S-parameter of the transmitted light (Fig. 3(a)). It also affects the transmission (Fig. 3(b)), which will be used for calculating hologram’s efficiency.

**Figure 4:**
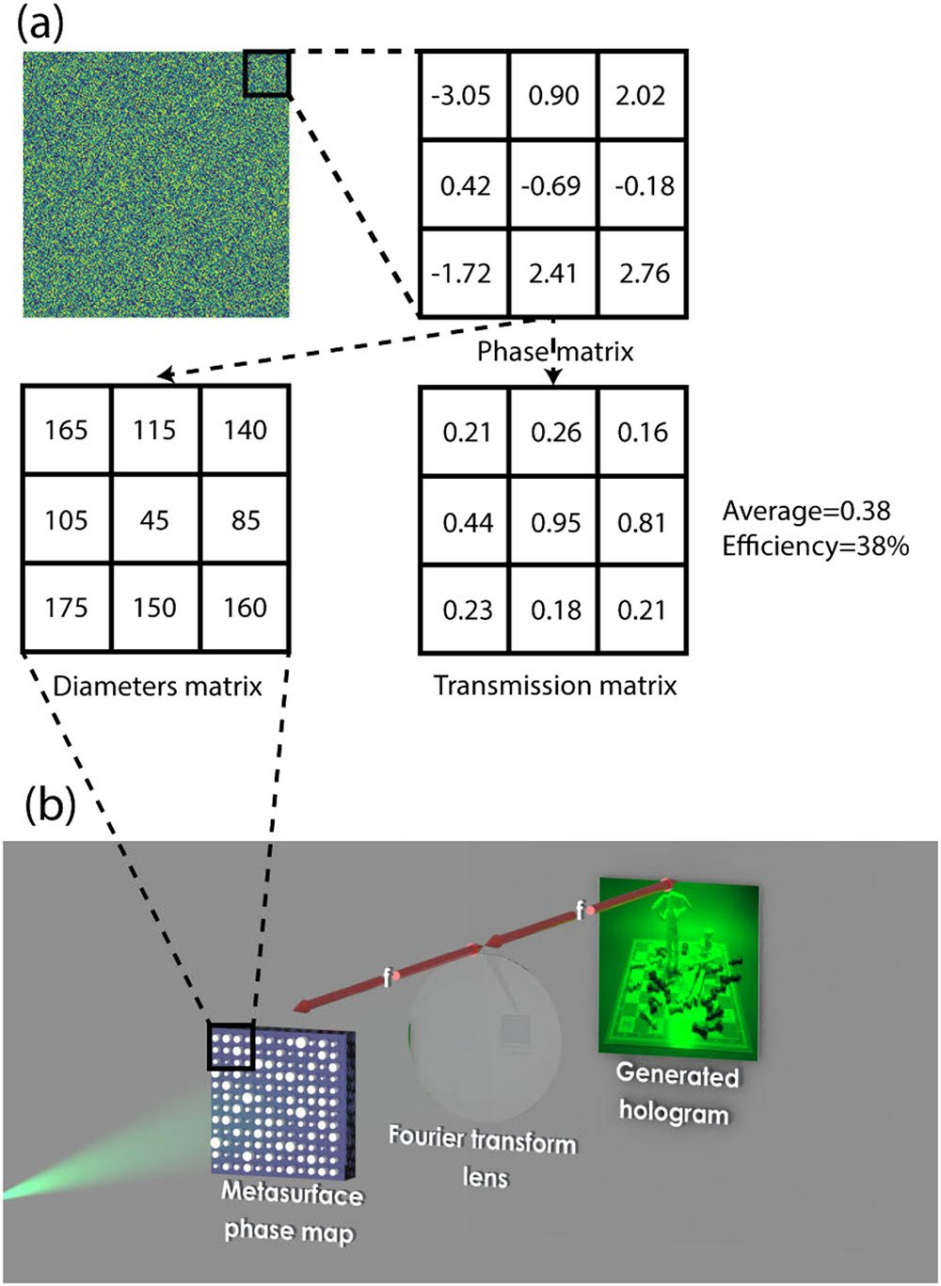
Procedure to generate holograms from metasurfaces. (**a**) Transforming phase map to phase matrix, diameter matrix and transmission matrix for constructing the final structure and calculating transmission efficiency. This is shown for a 3 × 3 pixel subsection of the phase map. (**b**) Converting phase map to hologram.

To generate the hologram, we need the phase map of our desired image. To find the phase map of a given image, we used an algorithm^43^ that creates a numerical phase map from a given image. Once we have the phase map, the next step is to construct the phase map by metasurfaces (Fig. 4(a)). The phase map is a matrix of numbers. We replace each phase by its corresponding diameter (Fig. 3(a)); this process yields a matrix of diameters by which we can construct an array of metasurfaces, and create the needed phase map and also calculate the efficiency of the hologram (Fig. 3(b)). The full phase map contains all the phases. A Fourier transform of the phase map generates a hologram (Fig. 4(b)). This procedure is done physically by using a Fourier transform lens^44^ or done numerically by applying a Fourier transform to the phase map matrix.

**Figure 5:**
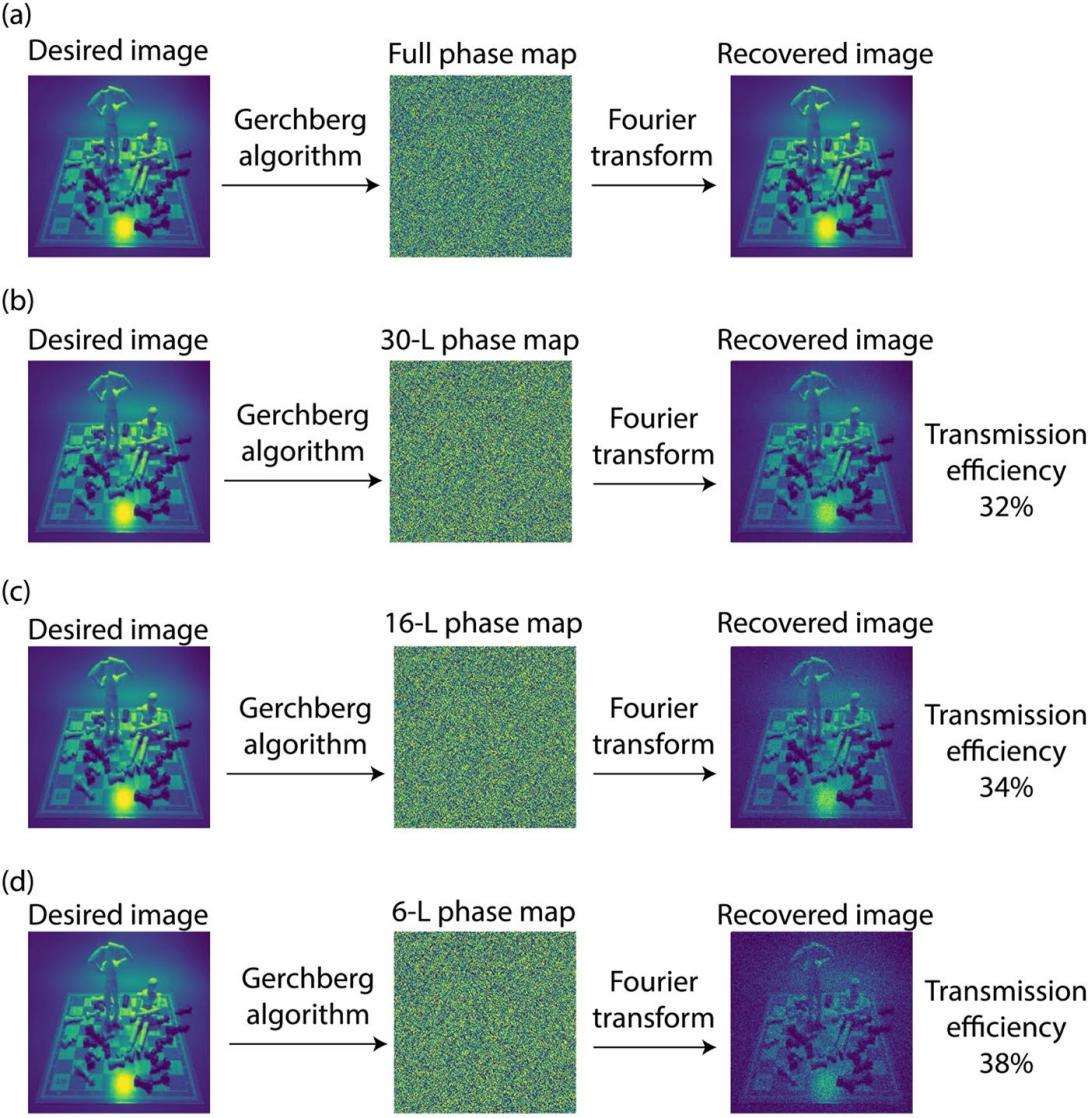
Numerically-generated holograms and transmission efficiencies of the structure found by DDQN. (**a**) Desired image and its full phase map, and recovered image from the full phase map. The desired image and its 30-level (**b**), 16-level (**c**) and 6-level (**d**) phase map and their corresponding images recovered by Fourier transform, and the calculated transmission efficiencies. Increase in the level of the phase map, increases the image quality and decreases the transmission efficiency.

To generate the whole phase map, we need all radii from 45 nm to 190 nm. However, fabricating radii with a 1 nm is precision is impossible in practice, so we chose only some of them. This process is known as an m-level phase map, in which m represents the number of chosen radii. For example, m = 6 means that the phase map has 6 levels or in other words only 6 radii. This procedure leads to loss of data and decreases the quality of the recovered image (Fig. 5(a–d)). Only 6 cylinders were used to calculate the final phase map and since the average of transmission power of those cylinders were higher than the average of transmission power of all the cylinders, the efficiency of 6-level phase map is higher in this case.

## Results

The simulations were done at 532 nm (green) to be compatible with most recent experimental work in the visible range^21,23,45^. The numerical simulations were performed in Lumerical and the machine learning codes are written in Python. A machine with a 16-core 3.40 GHz processor, 64 GB of RAM, and a NVIDIA GTX 1080ti GPU with 11GB DDR5X RAM was used. Although the number of states was ~5.7 billion, DDQN found the optimal results in only 2169 steps. As can be seen the model could find the results pretty fast. This may imply that the model just found this result by random guessing. As can be seen from Fig. 2 after step 400 the model predicts the actions just by learning and only 10 percent of the actions are done by guessing. It should be noted that there might be better answers than what the model found. So based on how long we let the model run, how the good the rival results are, initial conditions or complexity of the problem, it may take longer or shorter to find the optimum results.

It took a month for coding and running the model. The time is variable for different problems based on their complexity.

The final structure is as follows:

- Nano-disk material type (D_M): 19 (Indium phosphide) (Table 2)
- Thin film material type (F_M): 23 (SiO2 Glass)
- Grating material type (G_M): 23 (SiO2 Glass)
- Nano disk thickness (D_H): 190 nm
- Film thickness (F_H): 10 nm
- Grating thickness (G_H): 20 nm
- Grating coverage (G_C) in each unit cell: 0\%
- The spacing between disks (L): 70 nm
- Minimum transmitted power: 0.16

We can compute the efficiency of the hologram by calculating the average transmitted power from the phase map. We have the transmission for each of the radii (Fig. 3(b)), so by counting the number of each of the radii used in the phase map and calculating the average of transmission power, we can estimate the efficiency of the corresponding phase map (Fig. 5(a)). This is a rough estimation for two reasons. First the coupling between adjacent cylinders should be considered, and second, each image will have its own phase map and so different setup of cylinders is used for each image which results in different transmission efficiencies. So the only way to correctly find the transmission efficiency of a hologram is by fabricating it, and each image will have its own efficiency^21^. But this method gives us an approximate estimation of the average transmission efficiency as is shown in Fig. 4.

The computed transmission efficiency was 32% for a high-quality recovered image (Fig. 5(a)). Compared to the structures with the same properties (transmission type, polarization independent, and in visible regime) our proposed structure’s transmission efficiency is two times higher than^21^ with 17% transmission efficiency (theoretical) (6% experimental), and much higher than other similar work^24^ with <1% transmission efficiency(experimental). It should be noted that what we computed here is the total transmission efficiency (that is defined as the ratio of image intensity to the total power of incident light) and it shouldn’t be confused with diffraction efficiency (that is defined as the ratio of image intensity to the total power of hologram plane^24^). In diffraction efficiency, the source monitor is placed after the hologram (unlike the transmission efficiency in which the source monitor is placed before hologram) and so the efficiency is much higher compared to transmission efficiency, since the effect of hologram is neglected. A comparison of our hologram’s transmission efficiency with some of the previously reported results is shown in Table 3.

**Table 3:** Comparison of our hologram’s efficiency with some of the previously reported results.

Year	Ref.	Visible regime	Transmission type	Polarization Independent	Transmission Efficiency
2019	Ours	✓	✓	✓	32% (theo.)
2018	Yoon, G. *et al*.^21^	✓	✓	✓	17% (theo.), 6% (exp.)
2017	Huang, K. *et al*.^24^	✓	✓	✓	<1% (exp.)
2016	Xiong Li *et al*.^22^	✓	✓	✗	3.13% (theo.)
2013	Ni X. *et al*.^25^	✓	✓	✗	10% (exp.)
2016	Wang Li *et al*.^20^	✗	✓	✓	>90% (exp.)

## Conclusion

Here, we used double deep Q-learning to optimize a hologram structure to increase its efficiency. The DDQN model optimized the geometrical properties and also found the best material types for the structure. The hologram structure reported here is transmission type, works in the visible range and is independent of polarization. The previously reported structures with these properties had a maximum of 17% transmission efficiency, but our AI code could find a structure that had a 32% transmission efficiency while yielding a high-quality output.

## Data Availability

All data generated or analysed during this study are included in this published article (and its Supplementary Information files).

## References

[1] N. K. Kildishev AV, Boltasseva A, Shalaev VM (2012). Broadband light bending with plasmonic nanoantennas. Science.

[2] Sun S (2012). Gradient-index meta-surfaces as a bridge linking propagating waves and surface waves. Nature materials.

[3] Yu N (2011). Light propagation with phase discontinuities: generalized laws of reflection and refraction. science.

[4] Kim I (2018). Thermally robust ring-shaped chromium perfect absorber of visible light. Nanophotonics.

[5] Rana AS (2018). Tungsten-based ultrathin absorber for visible regime. Scientific Reports.

[6] Kim I (2018). Outfitting next generation display with optical metasurfaces. ACS Photonics.

[7] Lee T (2018). Plasmonic- and dielectric-based structural coloring: from fundamentals to practical applications. Nano Convergence.

[8] Li Z (2018). Full-space cloud of random points with a scrambling metasurface. Light: Science & Application.

[9] Aieta F (2012). Aberration-free ultrathin flat lenses and axicons at telecom wavelengths based on plasmonic metasurfaces. Nano letters.

[10] Ni X, Ishii S, Kildishev AV, Shalaev VM (2013). Ultra-thin, planar, Babinet-inverted plasmonic metalenses. Light: Science & Applications.

[11] Li G (2013). Spin-enabled plasmonic metasurfaces for manipulating orbital angular momentum of light. Nano letters.

[12] Mahmood N (2018). Polarisation insensitive multifunctional metasurfaces based on all-dielectric nanowaveguides. Nanoscale.

[13] Devlin RC, Khorasaninejad M, Chen WT, Oh J, Capasso F (2016). Broadband high-efficiency dielectric metasurfaces for the visible spectrum. Proceedings of the National Academy of Sciences.

[14] Lee G-Y (2018). Complete amplitude and phase control of light using broadband holographic metasurface. Nanoscale.

[15] Yoon G (2018). "Crypto-display" in dual-mode metasurfaces by simultaneous control of phase and spectral responses. ACS Nano.

[16] Li Z (2017). Dielectric meta-holograms enabled with dual magnetic resonances in visible light. ACS Nano.

[17] Ansari MA (2019). A spin-encoded all-dielectric metahologram for visible light. Laser and Photonics Reviews.

[18] Arbabi A, Horie Y, Bagheri M, Faraon A (2015). Dielectric metasurfaces for complete control of phase and polarization with subwavelength spatial resolution and high transmission. Nature nanotechnology.

[19] Chong KE (2016). Efficient polarization-insensitive complex wavefront control using Huygens’ metasurfaces based on dielectric resonant meta-atoms. Acs Photonics.

[20] Wang L (2016). Grayscale transparent metasurface holograms. Optica.

[21] Yoon G, Lee D, Nam KT, Rho J (2017). Pragmatic metasurface hologram at visible wavelength: the balance between diffraction efficiency and fabrication compatibility. ACS Photonics.

[22] Li X (2016). Multicolor 3D meta-holography by broadband plasmonic modulation. Science advances.

[23] Huang K (2015). Ultrahigh-capacity non-periodic photon sieves operating in visible light. Nature communications.

[24] Huang K (2017). Photon-nanosieve for ultrabroadband and large-angle-of-view holograms. Laser & Photonics Reviews.

[25] Ni X, Kildishev AV, Shalaev VM (2013). Metasurface holograms for visible light. Nature communications.

[26] Zheng G (2015). Metasurface holograms reaching 80% efficiency. Nature nanotechnology.

[27] Wang B (2016). Visible-frequency dielectric metasurfaces for multiwavelength achromatic and highly dispersive holograms. Nano letters.

[28] Qin F (2016). Hybrid bilayer plasmonic metasurface efficiently manipulates visible light. Science advances.

[29] Ding X (2015). Ultrathin Pancharatnam–Berry Metasurface with Maximal Cross-Polarization Efficiency. Advanced Materials.

[30] Liu D, Tan Y, Khoram E, Yu Z (2018). Training deep neural networks for the inverse design of nanophotonic structures. ACS Photonics.

[31] Peurifoy, J. *et al*. In *Frontiers in Optics*. FTh4A. 4 (Optical Society of America).

[32] Malkiel, I. *et al*. *Deep learning for design and retrieval of nano-photonic structures. arXiv preprint arXiv* **1702**, 07949 (2017).

[33] Peurifoy J (2018). Nanophotonic particle simulation and inverse design using artificial neural networks. Science advances.

[34] Sajedian I, Kim J, Rho J (2019). Finding the optical properties of plasmonic structures by image processing using the combination of convolutional neural networks and recurrent neural networks. Microsystems and Nanoengineering.

[35] So S, Rho J (2019). Designing nanophotonic structures using conditional-deep convolutional generative adversarial networks. Nanophotonics.

[36] So S, Mun J, Rho J (2019). Simultaneous inverse design of materials and structures via deep learning: Demonstration of dipole resonance engineering using core-shell nanoparticles. ACS Applied Materials & Interfaces.

[37] Ma W, Cheng F, Liu Y (2018). Deep-learning-enabled on-demand design of chiral metamaterials. ACS nano.

[38] Mnih V (2015). Human-level control through deep reinforcement learning. Nature.

[39] Sajedian I, Badloe T, Rho J (2019). Optimisation of colour generation from dielectric nanostructures using reinforcement learning. Optics express.

[40] Bukov M (2018). Reinforcement learning in different phases of quantum control. Physical Review X.

[41] Sajedian I, Zakery A, Rho J (2017). High efficiency second and third harmonic generation from magnetic metamaterials by using a grating. Optics Communications.

[42] Sutton, R. S. & Barto, A. G. *Reinforcement learning: An introduction*. (MIT, 1998).

[43] Gerchberg RW (1972). A practical algorithm for the determination of phase from image and diffraction plane pictures. Optik.

[44] Goodman, J. W. *Introduction to Fourier optics*. (Roberts and Company Publishers, 2005).

[45] Huang K (2016). Silicon multi-meta-holograms for the broadband visible light. Laser & Photonics Reviews.

